# Open questions in low oxidation state group 2 chemistry

**DOI:** 10.1038/s42004-020-00408-8

**Published:** 2020-11-06

**Authors:** Cameron Jones

**Affiliations:** School of Chemistry, Monash University, PO Box 23, Melbourne, VIC 3800 Australia

**Keywords:** Organometallic chemistry, Coordination chemistry

## Abstract

The chemistry of stable low oxidation state group 2 metal compounds was initiated in 2007 and has since expanded rapidly, yielding many surprises. Here the author outlines advances in the field and discusses some of the open questions and challenges that remain to be answered in coming years.

## The scene so far

Prior to 2007, the extensively developed chemistry of the group 2 (alkaline earth, Ae) metals had not been extended to the isolation of any stable molecular compound containing those metals in anything but the +2 oxidation state. This is despite related low oxidation-state chemistry of the *p*-block elements being well established over the preceding decades. With that said, a number of transient low oxidation-state Ae compounds, e.g., XMgMgX and ∙MgX (X = H or halide), had been studied in solid matrices at very low temperature (typically <10 K), or in the gas phase at low pressure. Moreover, a number of solid-state materials exhibiting bonding interactions between Ae metals had been described, e.g., superconducting MgB_2_. In addition, magnesium(I) compounds, e.g., ∙ MgNC, were detected in interstellar clouds^1^.

**Fig. 1:**
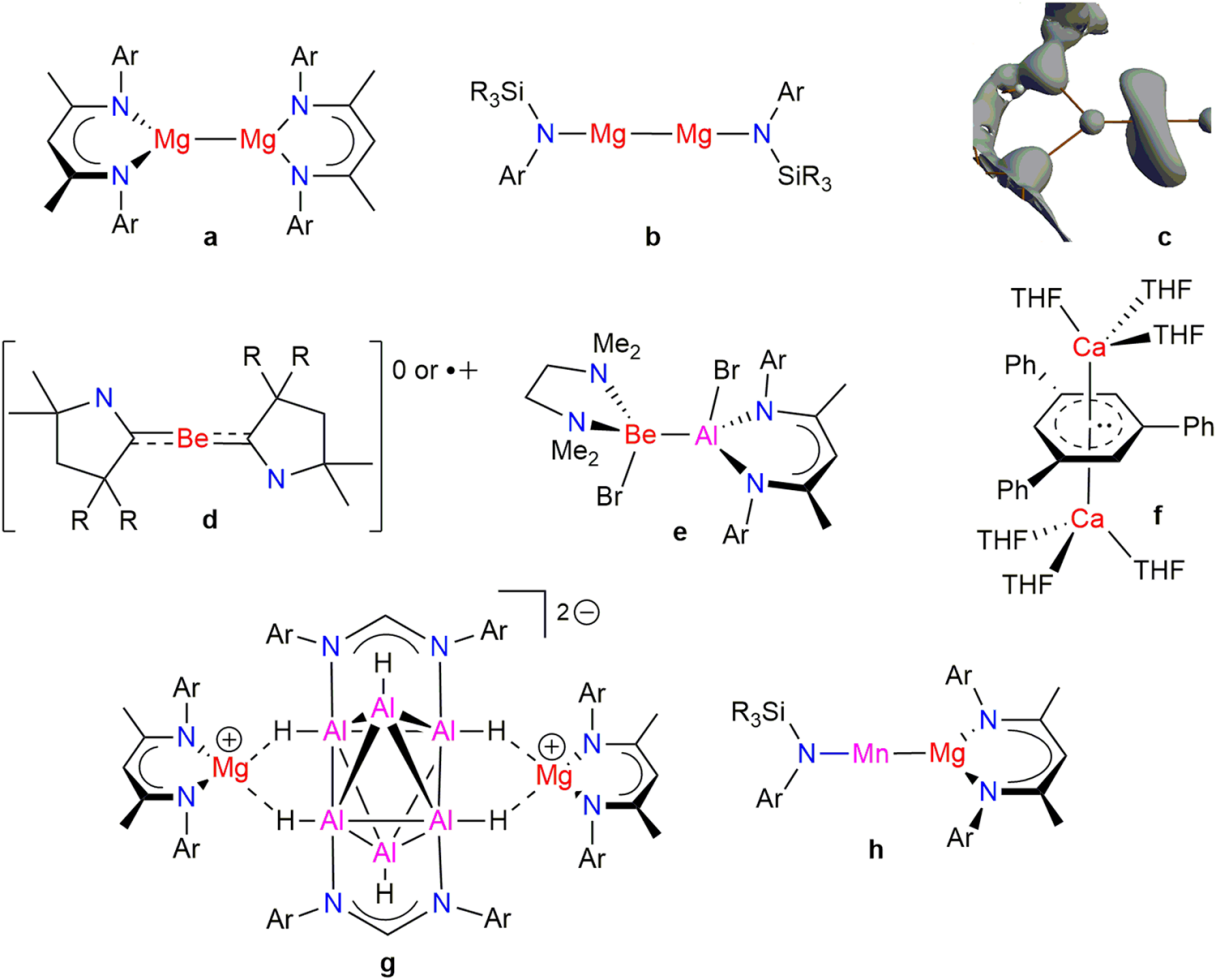
Reported low oxidation-state group 2 compounds and related metal–metal bonded complexes. Magnesium(I) systems (**a**, **b**) and a laplacian depiction of the non-nuclear attractor in the Mg–Mg bond of one of these (**c**, reprinted with permission from ref. ^5^. Copyright 2011 American Chemical Society), beryllium(0) and beryllium(I) complexes (**d**, **e**), a calcium(I) complex (**f**), a polyhedral aluminium(I) hydride cluster (**g**) and an unsupported Mn-Mg bonded complex (**h**) (Ar = bulky aryl, R = alkyl).

It is not surprising that stable low oxidation-state Ae compounds were late to arrive, given many computational studies showing that their disproportionation to elemental Ae metal and Ae^2+^ compounds is exothermic, and therefore thermodynamically favourable. This impediment was finally overcome in 2007 with the synthesis of the first magnesium(I) compounds, which were kinetically stabilised towards disproportionation by the incorporation of extremely bulky, chelating, anionic *N*-donor guanidinate or β-diketiminate ligands (e.g., see Fig. 1a)^2^. Since that time, more than 25 further examples of such compounds have come forward, in which the magnesium centres have coordination numbers of two, three or four (e.g., 2-coordinate, Fig. 1b)^3^.

Many of these magnesium(I) compounds are remarkably thermally stable (some decompose >300 °C) and are readily manipulated in the solid state or solution using standard Schlenk techniques. They possess Mg–Mg bonds, which theoretical and experimental studies initially described as being ‘normal’ covalent bonds (bond dissociation energies, BDEs, of ca. 45 kcal/mol)^1^. That is, the compounds were viewed as containing a central [Mg–Mg]^2+^ dication, exhibiting largely ionic interactions with bulky anionic amide ligands. The Mg–Mg *σ*-bonds are mainly associated with the rather diffuse highest occupied molecular orbital (MO) of the magnesium(I) compounds, whereas an empty *π*-type bonding orbital between the Mg centres is often associated with the lowest occupied MO or another low-lying unfilled MO. Experimentally, Mg–Mg bonds have been shown to be readily elongated (e.g., when coordinated by simple Lewis bases) and their lengths can lie over a remarkably wide range (ca. 2.8–3.2 Å)^4^. Computational studies revealed that this arises due to a very shallow bond elongation potential energy surface. More recently, computationally augmented experimental charge density studies have shown that Mg–Mg bonds actually do not possess classical covalent interactions, but instead contain a non-nuclear attractor (NNA), or nucleus-free local maximum of electron density, near their centre (Fig. 1c)^5^. To some extent, this explains the deformability and electronic diffuseness of Mg–Mg bonds. It is of note that magnesium(I) systems were the first stable molecular compounds to exhibit NNAs, which had previously only been calculated for some materials or unstable molecules, e.g., Na_2_.

Although stable magnesium(I) compounds have dominated low oxidation-state Ae chemistry since 2007, there has been sporadic progress with other related Ae systems since then. For instance, mononuclear cyclic alkylaminocarbene (CAAC) complexes of beryllium, with that element in the 0 or +1 oxidation state have been reported (Fig. 1d)^6,7^. These are stabilised by a considerable degree of Be→C *π*-backbonding of valence beryllium electron density to the redox non-innocent CAAC ligands. Recently reported compounds containing unsupported Be–Al covalent bonds can also be considered as ‘quasi’-Be(I) species, considering the very similar electronegativities of the metals involved (Fig. 1e)^8^. It should be noted that, in addition to the well-defined Mg–Mg compounds mentioned above, magnesium(I) halides, e.g., ∙MgBr and Mg_2_Br_2_, have been generated and studied in solution, but are only stable at temperatures <−40 °C^9^. Remarkably, reaction of ∙MgBr with KCp* (Cp* = C_5_Me_5_^−^) led to a black solid, which mass spectrometric and computational studies suggest contains a cluster anion, [Mg_16_Cp*_8_Br_4_K]^−^, which possesses 27 Mg–Mg bonds^10^. The only thermally stable molecular compound to contain one of the heavier Ae elements in a low oxidation state is a paramagnetic, formally calcium(I) inverse sandwich complex (Fig. 1f)^11^.

While of considerable fundamental interest, the high reactivity and unique properties of low oxidation-state Ae compounds lends them to synthetic, and other, applications. Not surprisingly, given their state of development, stable magnesium(I) compounds have dominated in this arena, although other systems have been studied. The very diffuse nature of the unusual Mg–Mg bonds in these compounds has seen them act as ‘molecular bottles’ for the storage of electrons, which can be delivered to organic and inorganic substrates in a controlled and stoichiometric manner, in the solution state. This has led to such compounds acting as specialised reducing agents in the formation of numerous compounds that cannot be accessed by the use of other, more traditional, reducing agents, e.g., KC_8_^12^. For example, in inorganic synthesis, magnesium(I) compounds have been used to prepare a multitude of low oxidation-state *p*- and *d*-block compounds, including unprecedented examples of metal–metal bonded species (e.g., see Fig. 1g, h^13,14^). In organic synthesis, a wide variety of reactions involving reductive coupling, cleavage and oligomerizations of small molecule substrates, in addition to catalytically relevant carbon–element bond activations, have been achieved^12^.

## Current and future challenges

**Fig. 2:**
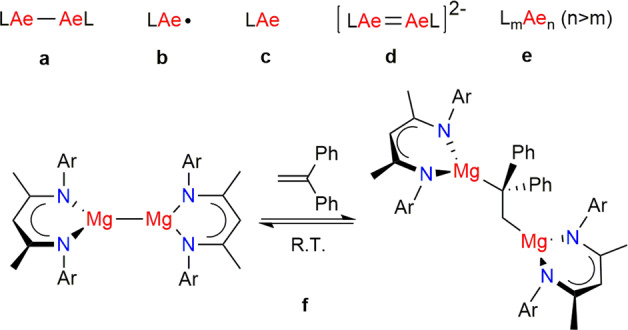
Target low oxidation-state group 2 compounds. Compounds with the Ae metal in the +1 oxidation state (**a**, **b**), 0 oxidation state (**c**, **d**) and mixed oxidation states (**e**). A reported room temperature redox reversible (Mg^I^/Mg^II^) chemical process (**f**). Ligands (L) will need to be extremely bulky, bi- or polydentate and exhibit a degree of structural rigidity, to avoid intramolecular bond activation processes.

Although rapidly developing, there is still much to achieve with respect to the fundamental and applications chemistry of low oxidation-state Ae compounds, just some pertinent examples of which will be discussed here. Perhaps the most obvious target molecules in the field are beryllium and heavier Ae analogues of known Mg–Mg bonded systems (Fig. 2a). Considerable efforts have already been made to access such systems, but to no avail. It is clear that new ligand types with specific steric and electronic properties will need to be designed, perhaps informed by computational studies, to stabilise these, and other, low oxidation-state Ae compounds. For example, although Be^I^–Be^I^ bonds should be quite strong (BDEs of ca. 70 kcal/mol), the small size of beryllium will require ligands small enough to accommodate such bonds, yet large enough to kinetically stabilise them toward disproportionation. In contrast, the calculated weak and very long heavier Ae^I^–Ae^I^ bonds (e.g., Ca^I^–Ca^I^ BDEs < 30 kcal/mol, bond length of ca. 3.8 Å^1^) will necessitate extremely large, rigid, probably tridentate, ligands to completely enshroud the Ae^I^–Ae^I^ bond, while not reacting (e.g., ligand C–H or C–C activations) with the very reducing Ae^I^ centres of the compound.

Another class of compound currently being pursued are stable monomeric radical compounds involving the Ae metals (Fig. 2b). Towards this end, reactions of known magnesium(I) compounds, LMg–MgL, with strong Lewis bases, e.g., N-heterocyclic carbenes (NHCs), have led to a considerable lengthening of the Mg–Mg bond (sometimes by >0.2 Å), or in some cases Mg–Mg cleavage, leading to the probable generation of transient, highly reactive magnesium(I) radicals, LMg^I^ ∙ ^1,4^. One valuable spin-off of this work is the fact that some mono-adduct complexes, e.g. LMg–Mg(NHC)L, have elongated and polarised Mg–Mg bonds, which have significantly enhanced abilities to activate small molecules in value adding synthetic processes^4^. Some success has also been had with the generation of transient magnesium(I) radical complexes *via* reduction of extremely bulky magnesium(II) halides, e.g., LMgI (L = very bulky ligand), although to date, stable magnesium(I) radicals remain elusive^15^. Ultimately, this will require more appropriately stabilising ligands, especially if heavier Ae^I^ radical species are to be isolated.

Of course, molecular compounds containing heavier Ae metals in the 0 oxidation state are valid objectives also (cf. Fig. 1d). Neutral mononuclear compounds of the type LAe^0^ (Fig. 2c) could conceivably be isolated where L is a bulky, neutral, polydentate, redox innocent or non-innocent ligand. Moreover, anionic dinuclear, doubly bonded Mg^0^, and other Ae^0^, systems, e.g., [LMg = MgL]^2−^_,_ can be envisaged to arise from the double reduction, and population of the empty π-bonding orbital of LAe–AeL compounds, by alkali metals or similar strong reductants. Other related synthetic targets that can be imagined are mixed-valence Ae compounds, [L_*m*_Ae_*n*_] (Fig. 2e, *m* < *n*, avge. Ae oxidation state < 1), which would possess more than one Ae–Ae bond, and ‘naked’ Ae atoms that are only bonded to other Ae atoms. Similar to the only reported, but ill-defined, example of such a compound, [Mg_16_Cp*_8_Br_4_K]^−^, these could shed light on the mechanism of formation of Grignard reagents, which after more than 100 years, is still unknown. Moreover, as molecular ‘cut outs’ of the bulk metal, they could be used as soluble models for the study of reversible hydrogen storage systems, such as the Mg/MgH_2_ couple.

The structural and bonding properties of newly developed Ae systems will be fascinating. To fully understand these properties, the compounds will need to be probed by a barrage of computational, spectroscopic, crystallographic and electrochemical techniques. Similar to previously developed systems, their further reactivity, especially with respect to the ‘transition metal-like’ activation of catalytically relevant small molecules (e.g., H_2_, CO, C_2_H_4_, CO_2_), unsaturates and C-X (e.g., X = C, H, F, etc.) bonds, will be powerful. Although many such activations have been achieved with stoichiometric amounts of Mg^I^–Mg^I^ bonded compounds^4,16^, incorporating those activation events into catalytic cycles in which a low oxidation-state Ae compound is an ‘on cycle’ redox catalyst remains a ‘grand challenge’ in s-block chemistry.

For redox catalysis involving Ae compounds to be achieved, the Ae compound would have to cycle between oxidation states (e.g., +1/+2 or 0/+2) in a near thermoneutral manner, and with a low kinetic barrier to the process. Although this might seem highly unlikely for these classically redox inert metals, hope comes from recent reports on facile, room temperature redox reversible insertions of alkenes into the Mg–Mg bonds of magnesium(I) compounds (Fig. 2f)^17^. Catalytic behaviour for Ae metals not only has relevance for chemical synthesis, but also for biological processes. For example, it has been calculated that phosphate group transfer, which is an essential function of enzymes such as DNA polymerases, requires the formation of a Mg^I^–Mg^I^ covalent bond for the enzyme to function^18^. In a similar vein, facile and reversible Ae^+^/Ae^2+^ redox processes have been experimentally observed for the simple graphite intercalated cations [(en)Ae^I^–Ae^I^(en)]^2+^ ↔ [Ae^II^(en)_2_]^2+^ (Ae = Mg, Ca, Sr or Ba, en = ethylenediammine)^19^. Materials such as this hold promise for use within electrodes of, e.g., magnesium ion batteries.

In conclusion, although the chemistry of stable low oxidation-state group 2 compounds has come a long way since its inception in 2007, there a many more appealing synthetic targets to pursue. The stabilisation of these targets will require considerable innovation and ingenuity on the part of chemists involved in the field. The potential rewards are, however, rich, with many new and unusual properties, bonding modes, and reactivity characteristics on offer. Ultimately, these will lead to valuable applications being found for low oxidation-state Ae compounds, which currently seem but a mirage on the horizon. The next 5–10 years will surely see such apparitions come within grasp.
